# Performance of Danaini larvae is affected by both exotic host plants and abiotic conditions

**DOI:** 10.1002/ece3.7821

**Published:** 2021-06-23

**Authors:** Pedro Paulo da Silva Ferreira, Daniela Rodrigues

**Affiliations:** ^1^ Programa de Pós‐Graduação em Ecologia Instituto de Biologia Universidade Federal do Rio de Janeiro Rio de Janeiro Brazil; ^2^ Laboratório de Interações Inseto‐Planta Departamento de Ecologia Instituto de Biologia Universidade Federal do Rio de Janeiro Rio de Janeiro Brazil

**Keywords:** *Asclepias curassavica*, *Calotropis procera*, *Danaus*, host plant origin, plant–herbivore interactions

## Abstract

The consequences of the introduction of invasive plants for the diet of herbivorous insects have been little explored in nature where, potentially, abiotic and biotic factors operate. In this study, we examined the larval performance of two Neotropical Danaini butterflies when using either a native or an exotic Apocynaceae species as host plant in both field and laboratory experiments. Hosts greatly differ in their amount of latex exudation and other physicochemical traits, as well as in the amount of evolutionary time they have interacted with herbivores. First, herbivore performance on the hosts was investigated under laboratory conditions. Larvae of both Danaini species took more time to develop on the exotic host; larval survivorship did not vary between hosts. Second, first instar survivorship on both hosts was evaluated in two field sites, one site per host. To do so, in both sites half of the larvae were bagged (protected against both abiotic and biotic factors) while the remainder were nonbagged (exposed). The interaction between larval exposure with the use of the exotic host reduced larval survival. We concluded that the combined effects of host plant traits and abiotic factors reduced survival of herbivores in field conditions. Therefore, the performance of herbivores when using hosts of different origins should be considered together with the multiple ecological factors found in natural environments, as these factors can modify the result of plant–herbivore interactions.

## Graphical abstract

We examined the larval performance of two Neotropical Danaini butterflies when using either a native or an exotic Apocynaceae species as host plant in both field and laboratory experiments. By employing both a bitrophic and a tritrophic perspectives, we showed that the use of an exotic host, combined with abiotic factors, negatively affected larval performance. To our knowledge, this is the first study that examines the performance of herbivorous insects on both native and exotic hosts both in the laboratory, where abiotic conditions are under control, as well as in the field, where multiple factors can act on herbivores, including natural enemies and abiotic factors.

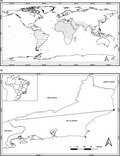

## 1 INTRODUCTION

In nature, herbivorous insects are faced with the decision to choose an appropriate host plant for oviposition and development of their offspring. In this context, exotic plants (i.e., plant species in a certain area whose presence is due to human intervention, intentional or not; see Pyšek et al., 2004) are potential resources for native herbivores. Many studies have examined the performance of native herbivores on native versus exotic host plants under controlled conditions, pointing out contrasting results; see Bezemer et al. (2014) for a review. Native species are defined as those that have evolved in a certain area or reached it by natural processes without human intervention (intentional or not; see Pyšek et al., 2004). In some cases, exotic plants represent a suitable resource for the offspring, improving the fitness of the herbivores that feed on them (Harvey, Biere, et al., 2010). In other cases, herbivore survival is reduced (Keeler & Chew, 2008) or other performance traits, such as development time and individual body size, are compromised (White et al., 2008). Thus, the introduction of exotic plants can directly affect the abundance and performance of native herbivores, and indirectly affect the structure of native herbivore–plant interactions and food webs (Pearson, 2009; Vilà et al., 2011).

The response of herbivores to introduced plants depends on factors such as feeding preferences, as well as physiological or behavioral adaptations to plant traits. Several studies have examined the bottom‐up selection factors imposed by exotic host plants on native herbivores. To do so, researchers have employed an experimental design that allows top‐down forces and abiotic factors to be excluded or fixed, thus isolating the bottom‐up factors. For example, Cogni (2010) carried out a common garden experiment to examine how the introduction of an exotic host affected a native specialist herbivore's preference and performance; DiTommaso and Losey (2003) and Mattila and Otis (2003) performed choice tests to evaluate oviposition site selection as well as larval feeding preference and performance of the monarch butterfly *Danaus plexippus* (Linnaeus, 1758) in the presence of both native (*Asclepias syriaca* L.) and invasive species (*Vincetoxicum* spp.) hosts. However, studies investigating the use of exotic plants and their effects on native insect–plant interactions under field conditions—where top‐down, bottom‐up, and abiotic factors can operate—remain scarce.

Under field conditions, multiple factors can interact and thereby affect larval mortality (Zalucki et al., 2002). For example, when feeding on host plant species on which larval development time was delayed, early instars of the cabbage white butterfly *Pieris rapae* (Linnaeus, 1758) suffered more attacks from a braconid parasitoid (Benrey & Denno, 1997). Indeed, top‐down forces have a great impact on immature insects’ populations (Cornell & Hawkins, 1995; Montllor & Bernays, 1993). Additionally, microclimate may be critical for the survival of early‐instar Lepidoptera that are small in size and limited in movement (Zalucki et al., 2002). Some studies have reported that wind and rainfall are among the major factors causing mortality in eggs (Kyi et al., 1991; Rahman et al., 2019) and newly hatched larvae (Caldas, 1992; Harcourt, 1966; Kyi et al., 1991), and light intensity can affect larval performance through its effects on the primary and secondary metabolism of host plants (Manuwoto & Scriber, 1985). Therefore, in field conditions a combination of factors may affect the interaction between herbivores and their host plants.

To better understand the effects of exotic plants on native herbivores, it is important to investigate how these interactions occur in the field, where these organisms are affected by multiple biotic (such as the presence of natural enemies) and abiotic (such as environmental conditions) factors (see Harvey et al., 2010). In this study, we investigated the use of a native milkweed with worldwide distribution and a non‐native and recently introduced Apocynaceae by two species of Neotropical Danaini, *Danaus erippus* (Cramer, 1775) and *Danaus gilippus* (Cramer, 1775) (Lepidoptera: Nymphalidae). Larvae of *Danaus* butterflies are oligophagous, that is, they feed uniquely on hosts belonging to Apocynaceae (Ackery & Vane‐Wright, 1984; Beccaloni, 2008; Smith, 2014). In the laboratory, the larval performance of these danaines on the above hosts was examined and compared. Additionally, we employed a well‐established method in the literature, which consists of using protection treatments against both natural enemies and abiotic factors (= bagging) to examine which of the above factors affect early instars under field conditions. Hosts greatly differ with respect to the amount of latex exuded upon damage and other physicochemical attributes, as well as in the evolutionary time of interaction with native herbivores—a century for the exotic host against a million‐year time scale for the native host (Ferreira, 2017). In this context, we predicted that Danaini larvae would perform poorly when using the exotic host plant in comparison with the native one, both in the laboratory and under field conditions.

Over recent decades several studies of the performance of butterfly species on native versus exotic or novel versus non‐novel hosts have been conducted (e.g., Bowers et al., 1992; Camara, 1997; DiTommaso & Losey, 2003; Forister et al., 2009; Gillespie & Wratten, 2011; Harvey, Biere, et al., 2010; Mattila & Otis, 2003; Rahman et al., 1985; see Bezemer et al., 2014 for a review). Such studies were mostly performed in the laboratory (i.e., under controlled conditions) or under seminatural conditions (greenhouse and insectary). In addition, the bitrophic perspective predominated in these studies, and suggestions regarding the importance of the third trophic level affecting the outcomes of the interactions between hosts plants and herbivores were made (e.g., Bowers et al., 1992; Camara, 1997; Forister et al., 2009; see Harvey, Bukovinszky, et al., 2010). Top‐down effects were taken into account in a few studies, with emphasis on tests using parasitoids under controlled or semicontrolled conditions (Fortuna et al., 2013). In general, exotic or novel plants have negative effects on the performance and fitness of native herbivores, see Vilà et al. (2011) for a review, while native plants accumulate higher herbivory loads when compared to introduced congeners (Liu & Stiling, 2006). Taken together, these studies identified the duration of interaction as an important factor mediating the performance of herbivorous insects on host plants, leading us to think that exotic plants impose greater barriers to herbivory in native herbivores when compared to their native, non‐novel counterparts. As a consequence, to our knowledge this is the first study that examines the performance of herbivorous insects on both native and exotic hosts both in the laboratory, where abiotic conditions are under control, as well as in the field, where multiple factors can act on herbivores, including natural enemies and abiotic factors. We defined performance according to Thompson (1988), that is, a composite term that includes several traits as for example larval survival and development time, as well as adult size.

## 2 MATERIALS AND METHODS

### 2.1 Study system

**FIGURE 1:**
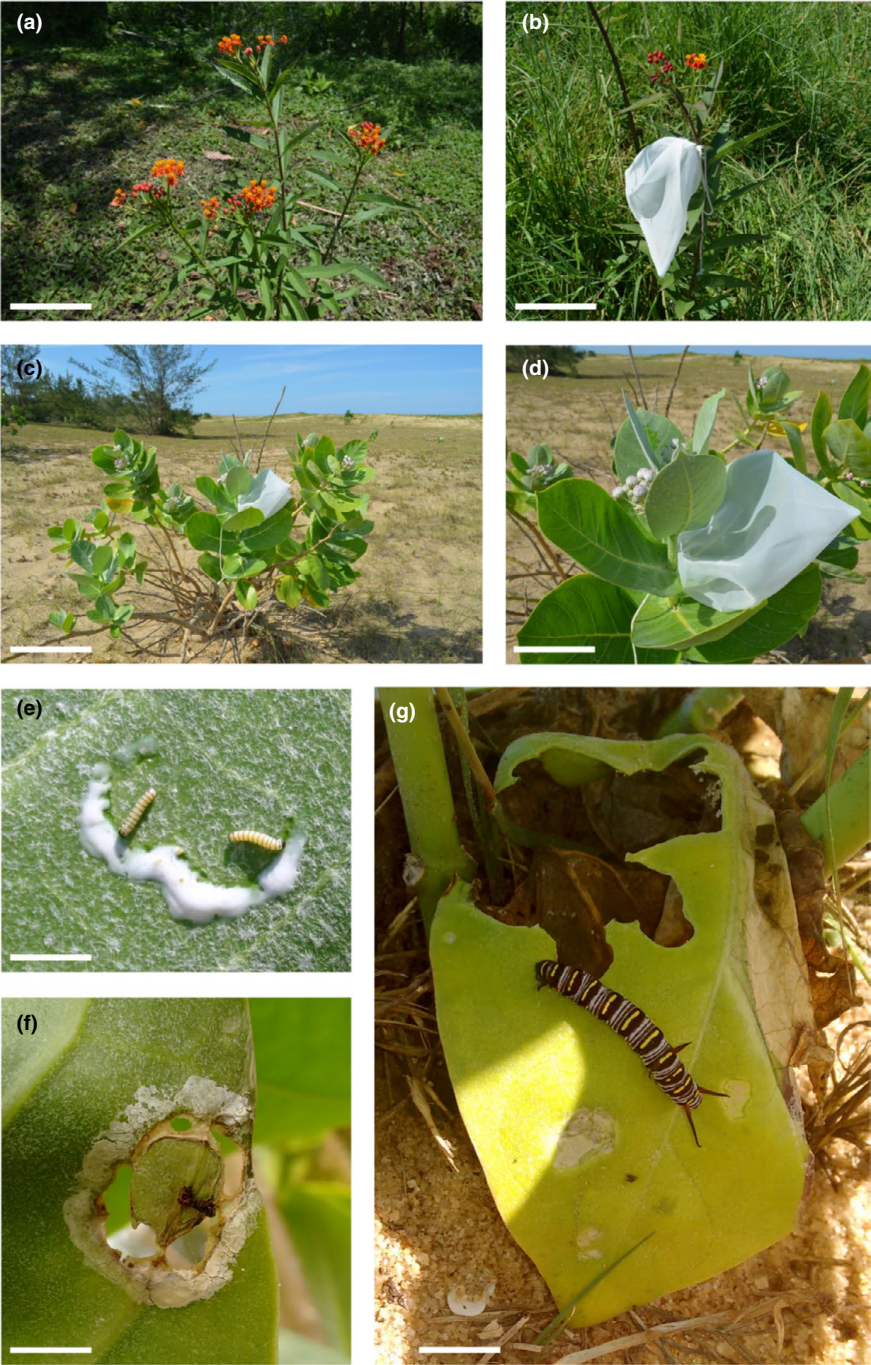
Study system. *Asclepias curassavica* at Itatiaia county, RJ (a). Individual of *A. curassavica* with a bagged leaf (b). *Calotropis procera* at Restinga do Xexé, Campos dos Goytacazes county, RJ (c). Note the bagged leaf. Bagged leaf of *C. procera* (d). First instars of *D. erippus* after feeding on an exposed leaf of *C. procera* (e). First (f) and last (fifth) (g) instars of *D. gilippus* on an exposed leaf of *C. procera*. Note the circular trenching and latex release in (e and f). Scale bars: 15, 10, 50, 15, 0.5, 0,5, and 1.6 cm, respectively

**FIGURE 2:**
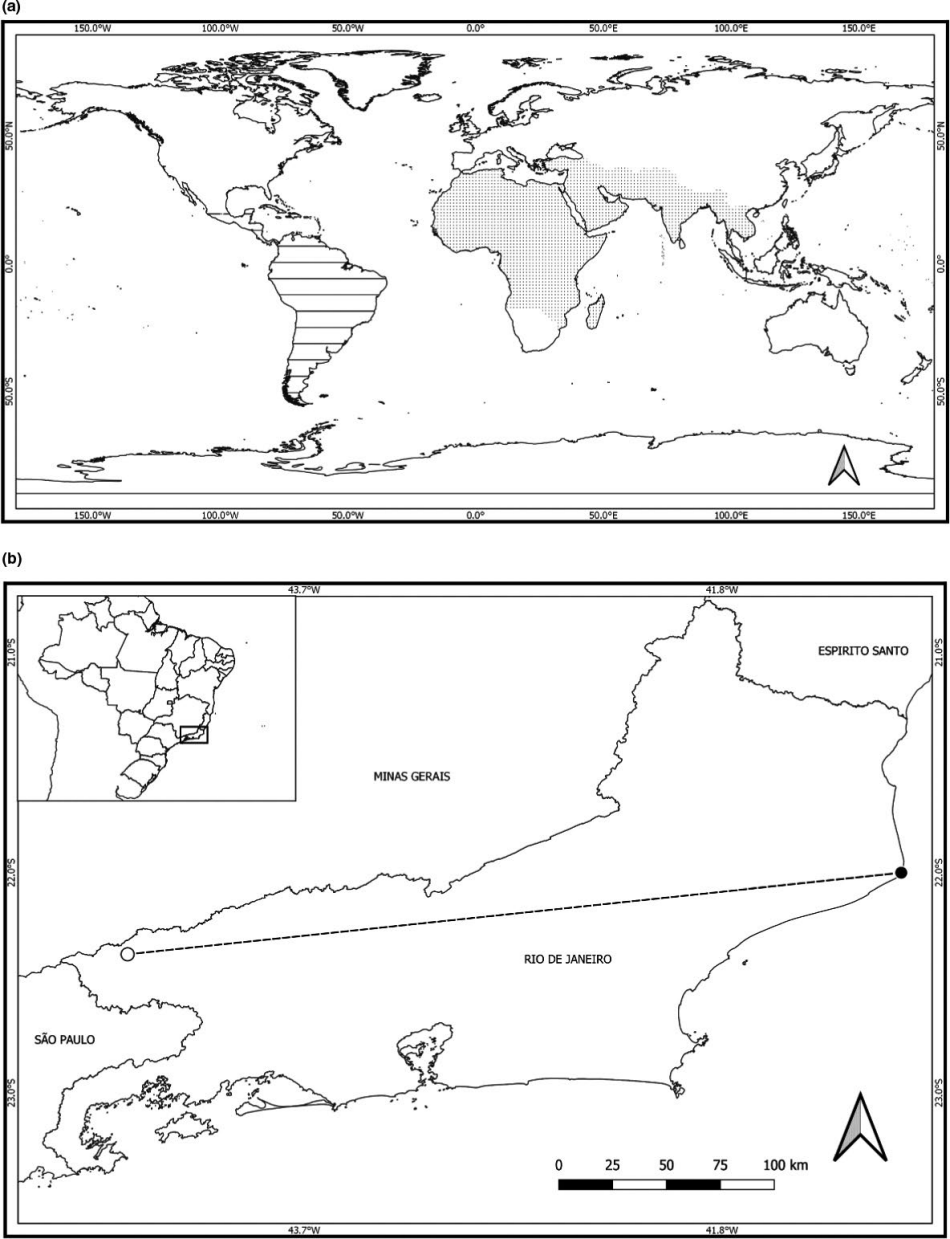
Original distribution of host plant species used in this study—*A. curassavica* (dashed area) and *C. procera* (shaded area)—according to records from the literature (see main text) (a). Map of Rio de Janeiro State highlighting the municipalities where field experiments were carried out for *A. curassavica* (Itatiaia county, open circle) and *C. procera* (Campos dos Goytacazes county, closed circle); the dashed line indicates the distance between these locations; the black square indicates the Brazilian territory as well as the location of Rio de Janeiro State (b). Maps were built using QGIS software (QGIS Development Team, 2019)

*Asclepias curassavica* L. (Apocynaceae), known as tropical milkweed, is a 1‐m‐tall herbaceous plant (Figure 1a,b), being either perennial or annual. *Asclepias curassavica* is considered native to the tropical Americas, and it has been introduced in other regions with a tropical and subtropical climate, and is now distributed worldwide (Holm et al., 1997; POWO, 2019; Flora do Brasil, 2020; see its original distribution in Figure 2a). *Asclepias curassavica* is reported to be the most abundant member of the Apocynaceae in South America (Bollwinkel, 1969) and is the main host of *Danaus* butterflies from South America (Beccaloni, 2008). *Asclepias curassavica* exudes small amounts of latex when damaged (Agrawal & Fishbein, 2006; Zalucki et al., 2012).

*Calotropis procera* (Aiton) R. Br. (Apocynaceae), popularly known as Apple of Sodom, is a perennial, erect, and slightly branched shrub (Figure 1c,d). *Calotropis procera* is native to tropical Africa, Madagascar, the Arabian Peninsula, and Southwest Asia (Rahman & Wilcock, 1991) (Figure 2a). *Calotropis procera* has recently increased its distribution to all the tropical regions of the world, especially arid and semiarid regions (Brandes, 2005). Due to its rapid establishment and growth, this plant species occurs at high density and is considered invasive in the Americas and Oceania (Instituto Horus 2016), commonly invading areas after disturbance (Brandes, 2005; Rangel & Nascimento, 2011). Its introduction to Brazil took place in the state of Pernambuco in 1900s, for ornamental purposes (Instituto Horus 2016). As it is easily dispersed by the wind and has a high establishment capacity, *C. procera* has spread along the coast reaching the north of Rio de Janeiro State (Rangel & Nascimento, 2011). Like other members of the Apocynaceae, *C. procera* is a latex producer, which is easily observed when its stem and leaves are damaged.

*Calotropis procera* is reported to be a host of Neotropical *Danaus* species, including *D. erippus* (Figure 1e), *D. gilippus* (Figure 1f,g), and *Danaus eresimus*, the soldier butterfly (Beccaloni, 2008; Fernandes et al., 2020). Both *A. curassavica* and *C. procera* are the most representative host plants of *Danaus* butterflies from eastern regions of Brazil, including Caatinga and Atlantic Forest areas. *Danaus erippus*, known as the southern monarch butterfly, is a sister species of the world‐renowned monarch butterfly—*D. plexippus*—and is restricted to South America. *Danaus gilippus*, the queen butterfly, occurs from southern North America, through Central America and the Antilles, to tropical regions of South America (Ackery & Vane‐Wright, 1984). Southern monarchs and queens are the only species from the *Danaus* genus consistently found in areas of the Atlantic Forest biome, as well as sharing the same hosts.

### 2.2 Larval performance under controlled conditions

Seeds of *A. curassavica* and *C. procera* were collected from several localities in southeastern Brazil and planted in plastic pots (35 cm diameter) containing a mixture of organic substrate (garden soil) and sand (2:1). Pots were placed in a greenhouse at the Horto da Prefeitura Universitária at the Universidade Federal do Rio de Janeiro, Rio de Janeiro State, southeastern Brazil. Eggs of *Danaus* spp. were collected from the field, in the vicinity of the Reserva Ecológica de Guapiaçu in Cachoeiras de Macacu municipality, Rio de Janeiro State (22º 27’ 10.30’’ S, 42º 46’ 13.05” W; 34 m a.s.l.). Due to the strong dispersal abilities of both plant and animal species included in this study, it was assumed that the individuals collected in different localities were not far enough from one another for local adaptations to occur. For example, *D. erippus* adults are able to perform migrations on a continental scale, which is evidence of the high dispersal capacity of this species (Malcolm & Slager, 2015).

In order to investigate herbivore performance under laboratory conditions, individuals of *A. curassavica* and *C. procera* were at least 6 months old and had a minimum of six intact, fully expanded leaves to assure ad libitum feeding. Larvae were individually assigned to potted plants, and each replicate was isolated by a white voile screen and taken to a climate‐controlled room (25 ± 1°C; 250 μmol.m^‐2^.s^‐1^ PAR; 14‐hr light: 10‐hr dark; 60%~80% RH). The following treatment combinations were used to measure larval performance on each host plant species: *D. erippus* on *A. curassavica*; *D. erippus* on *C. procera*; *D. gilippus* on *A. curassavica*; and *D. gilippus* on *C. procera* (*N* = 10/Danaini species/host plant species). Between 0 and 12 hr after hatching, larvae were gently placed on the respective host plant with a thin brush. Each larva was inspected daily in the morning; survivorship and instar were recorded. When larvae were close to pupation, they were transferred to plastic pots containing leaves of their respective host plant. The stems (in the case of *A. curassavica*) and petioles (in the case of *C. procera*) were kept in 1.5‐ml Eppendorf^®^ tubes filled with water. Each pupa was kept in the plastic pot until emergence. Development time was counted from egg hatching to emergence of the adult from the pupa. After emergence, the adult's forewing length was measured using digital calipers (Mitutoyo^®^); the sex was also recorded. The measure of survival of both Danaini species on each host was obtained from the percentage of individuals that reached the adult stage.

### 2.3 Larval survival under field conditions

In order to understand which factors—host plants, natural enemies and/or abiotic influences—act on populations of *Danaus* spp. under field conditions, larvae of *D. erippus* were monitored during the first instar on both host plants. This experiment was carried out using *D. erippus* only, because this species is considerably more abundant in comparison with *D. gilippus* (see Appendix S1). Because *C. procera* has spread in the Restinga areas of Rio de Janeiro State—an ecosystem in which *A. curassavica* is not expected to occur—we failed to find a place where the hosts co‐occur. As a consequence, the experiment was carried out in two localities in the state of Rio de Janeiro, southeastern Brazil, these being 365 km apart from one another (Figure 2b). The two localities above are located in the Atlantic Forest biome.

From 21 to 25 March 2016, fourteen individuals of *C. procera* were selected in the Restinga do Xexé, Campos dos Goytacazes municipality, Rio de Janeiro State (21º 59’ 58.03” S, 40º 59’ 06.85” W; 3 m a.s.l.). Individuals of *C. procera* were ca.1 m tall to allow the monitoring of larvae. A pair of newly hatched larvae of *D. erippus* was placed on each individual plant. These larvae were gently placed on opposite leaves positioned in the apical third of the shoot using a thin brush. One larva of each pair was isolated with its corresponding host leaf using a transparent voile bag, which makes it possible to monitor the larvae and prevents the access of predators and parasitoids (protection treatment; *N* = 14) (Figure 1d). The other larva did not receive any type of protection, remaining exposed on the host leaf (nonbagged treatment, *N* = 14). In order to minimize possible microenvironmental variation present in the Restinga, the position of the leaves corresponding to a given treatment was alternated, since the study area has the Atlantic Ocean on one side (about 100 m from its edge) and a unpaved road on the other side (about 50 m from its edge). As a consequence, only one leaf per individual plant was bagged, so that the remaining leaves remained exposed (Figure 1b–d). The voile is slightly transparent, so that we assumed that leaves were able to perform photosynthesis at the time period leaves were bagged (48 hr). In addition, the air temperature and relative humidity (mean, maximum, and minimum) of leaves from both treatments were measured and compared before the experiment commenced (*N* = 10 leaves/treatment; Appendix S1). No significant differences in these parameters were found between exposed and bagged leaves, showing that the voile tissue did not affect leaf quality and allows similar microclimatic conditions compared with the outdoor environment.

From 8 to 10 April 2016, the same experiment was performed on the native host plant *A. curassavica*, in a pasture area in which this host is very common (Figure 1b). As a consequence, the field experiment was performed in both localities with a time lag of 2 weeks during early fall and a time at which caterpillars abound in both tropical and subtropical regions (e.g., Rodrigues & Moreira, 2004).

The study area was located in Itatiaia municipality, Rio de Janeiro State (22º 22' 11.58" S, 44º 30' 10.41" W, 505 m a.s.l.). We established thirteen replicates (pairs) of each treatment, as described above for *C. procera*. However, due to livestock grazing at the site, three replicates of the protection treatment and one of the nonbagged treatment were lost during the night of 8 to 9 April, leaving ten and twelve larvae, respectively. As a consequence, the next night we removed all larvae from the treatments. Larval and bag removal occurred at 1,800 hr, and treatments were assigned again on the same leaves at 0,700 hr on 10 April 2016. Night removal of larvae was not an issue, as the expected natural enemies are visually orientated, that is, diurnal (Koch et al., 2003; Zalucki & Kitching, 1982).

Every time two larvae hatched, we set up a new replicate for the experiment. All larvae were followed for at least 48 hr or until second instar molting. In other words, only the first instars were used in the experiment, since 70% of Lepidoptera mortality occurs at this stadium (Zalucki et al., 2002; Zalucki & Kitching, 1982; Zalucki, Malcolm, et al., 2001). The larvae were observed through instantaneous observations (Martin & Bateson, 2007) performed at intervals of 30 min, between 0,800 hr and 1,700 hr. When observing larvae in the bagged treatment, bags were not removed. During the observations, we recorded larval presence, absence, or mortality, as well as feeding, latex exudation, predation, or parasitism events. As the absence of larvae can occur due to wind dispersal or attack/predation in the nonbagged treatment, the dependent variable considered was the larval presence in a given treatment. Therefore, larval presence was our proxy of survival under field conditions.

### 2.4 Statistical analysis

Larval survival under laboratory conditions was compared through Fisher's exact test between host plants (fixing the Danaini species) and Danaini species (fixing the host plant). Larval development time and forewing length were analyzed for their normality (Shapiro–Wilk test) and homoscedasticity (Bartlett's test). Forewing length for both Danaini species showed normal distribution and was compared between host plants using unpaired Student's *t* tests. Larval development time did not show normal distribution (for *D. erippus* reared on *A. curassavica* and *D. gilippus* on both hosts) and was compared between host plants using the Mann–Whitney test. Tests were performed using GraphPad Prism 8.3.1 software.

To examine which factors determine larval survival on host plants under field conditions, the variables were combined into generalized linear models. Host plant species (*A. curassavica* or *C. procera*) and treatment (bagged or nonbagged) were considered as fixed variables and larval survival as a response variable (binomial distribution). We tested models considering the effect of each fixed variable, as well as the additive effect of both variables and their interaction. In order to determine which model best explains the results, we performed model selection using the Akaike information criterion (AIC) and the “bbmle” package. Models were considered to be equally supported if the difference in AIC was less than two units (Burnham & Anderson, 2002). Tests were performed in the R environment (R Development Core Team, 2013) using R Studio software (v 1.2.1335).

## 3 RESULTS

### 3.1 Larval performance under controlled conditions

For both Danaini species, survival did not differ between hosts (Fisher's exact test; *D. erippus*, *p* = .5820; *D. gilippus*, *p* = 1.000) (Table 1); a similar pattern was observed when the Danaini species were compared on the same host (Fisher's exact test; *A. curassavica*, *p* = 1.000; *C. procera*, *p* = .5820). Development time was significantly higher for both *Danaus* spp. when reared on the non‐native milkweed *C. procera* (Mann–Whitney test; *D. erippus*, *p* = .0007; *D. gilippus*, *p* = .0010). Based on forewing length, *D. gilippus* adults reared on *C. procera* were significantly larger compared with those reared on *A. curassavica*; no differences were found in *D. erippus* (Student's *t* test; *D. gilippus*, *p* = .0439; *D. erippus*, *p =* .2341). The sex ratio remained close to 1:1 for *D. erippus* when reared on both host plants and for *D. gilippus* reared on *A. curassavica*. However, the proportion of males was higher (approximately 2:1) in *D. gilippus* reared on *C. procera*.

**TABLE 1:** Performance traits of *D. erippus* and *D. gilippus* under controlled conditions (*N* = 10/Danaini species/host plant) (mean ± standard error)

Danaini species	*Danaus erippus*		*Danaus gilippus*		Statistical analysis
Host plant	*Asclepias*	*Calotropis*	*Asclepias*	*Calotropis*	
Survivorship (%)	90	70	100	90	Fisher's Exact test
Development time (days)	13.00 ± 0.16^a^	17.43 ± 0.99^b^	14.20 ± 0.20^a^	16.33 ± 0.44^b^	Mann–Whitney test
Forewing length (mm)	47.06 ± 0.49	46.06 ± 0.65	41.53 ± 0.45^a^	42.67 ± 0.22^b^	Unpaired Student's *t* test

Note

For each Danaini species and performance trait, different letters denote significant difference between host plants (*p* < .05).

### 3.2 Larval survival under field conditions

**FIGURE 3:**
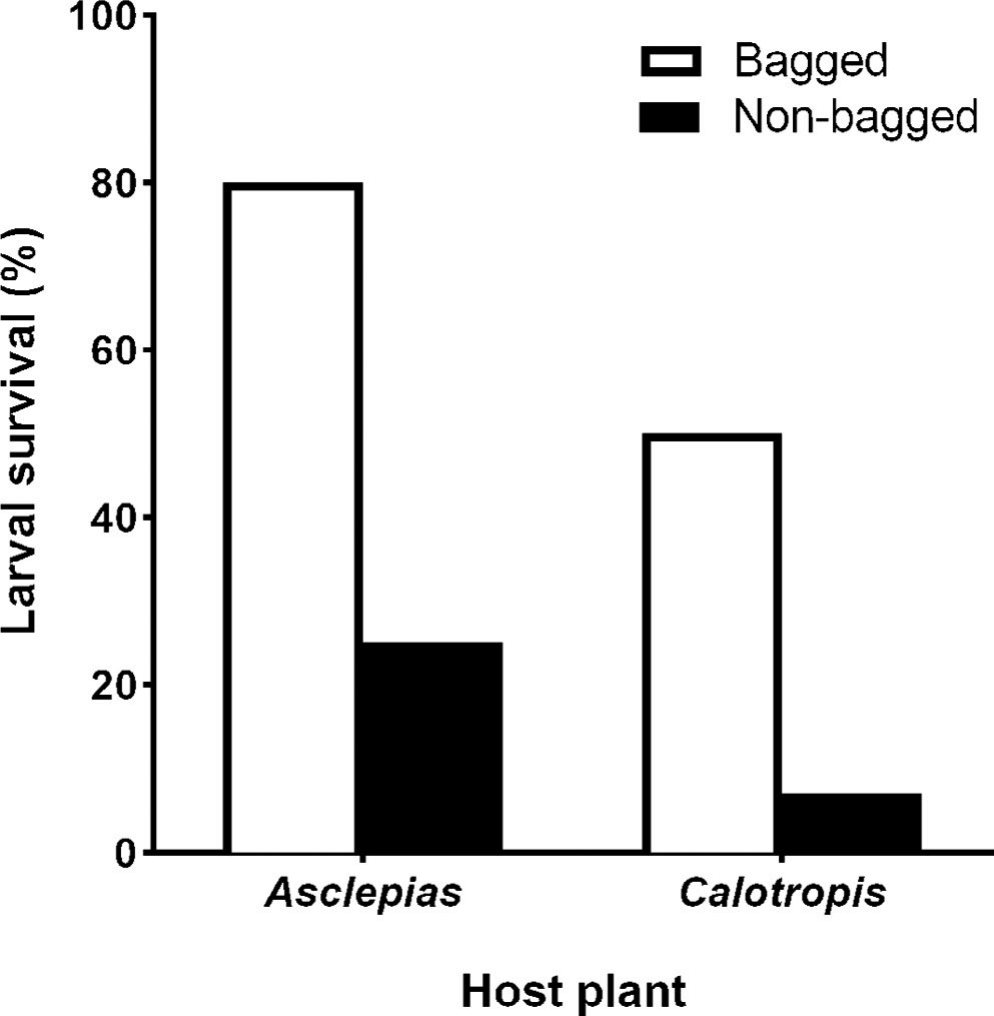
Larval survival of first instars of *D. erippus* under field conditions, assigned to either bagged or nonbagged treatments on both native (*A. curassavica*) and exotic (*Calotropis* procera) host plants

Larvae of *D. erippus* had higher survival on both hosts in the protection treatment, as well as when placed on the native host *A. curassavica* compared with the non‐native *C. procera* (Figure 3). The model that best explained larval survival includes the additive effect of "host plant" and "treatment" factors (Table 2). When the fixed variables were analyzed separately in this model, nonbagged treatment significantly reduced larval survival (*β* = −2.5204; *p* = .0011), while *C. procera* showed no significant effect (*β* = −1.4165; *p* = .0603). The model that includes only the “treatment” factor was also supported in the model selection (ΔAICc <2.0).

**TABLE 2:** Model selection performed through Akaike information criterion, considering “host plant” (*A. curassavica* or *C. procera*) and “treatment” (bagged or nonbagged) as predictors, and larval survival as response variable

Model	AIC_c_	Δ_i_	ω_i_	*K*	Deviance
Host plant + treatment	16.16	0.00	0.21	3	<0.01
Treatment	18.12	1.96	0.21	2	3.96
Host plant * treatment	18.16	2.00	0.57	4	<0.01
Host plant	28.04	11.88	0.00	2	13.88

Note

Variables were combined with (*) and without (+) interaction. Δ_i_: difference between AICc value; ωi: model weight; *K*: number of parameters.

Although predatory wasps from the genus *Polistes* were seen foraging around both host plants, no predation events were observed. Larval mortality could be attributed to latex exudation in some cases: 21.4% of larvae observed in *C. procera* (three from the nonbagged treatment and three from the bagged treatment) and 4.5% in *A. curassavica* (one from the bagged treatment). Other factors that caused mortality were desiccation and/or falling off the host plant due to the occurrence of wind: 35.7% of the larvae observed in *C. procera* (eight from the nonbagged treatment and two from the bagged treatment) and 40.9% in *A. curassavica* (eight from the nonbagged treatment and one from the bagged treatment).

## 4 DISCUSSION

Survival of immature *D. erippus* and *D. gilippus* did not differ between native and exotic host plants under laboratory conditions. On the contrary, feeding on the exotic host plant caused an increase in the larval development time of both butterfly species. The slower growth rate of *D. erippus* and *D. gilippus* on *C. procera* could reflect larval energy expenditure to handle large amounts of latex that this exotic host releases. Danaini larvae exhibit sabotaging behavior of latex defenses before feeding (see a review in Dussourd, 2017), and latex exudation during sabotaging can pose a risk to larval survival under field conditions (e.g., Zalucki Brower & Alfonso, 2001; Zalucki, Malcolm, et al., 2001; Zalucki et al., 2012; see Figure 1e,f). Indeed, the trade‐off between growth and mortality can be caused by different reasons, from physiological to ecological (see Mangel & Stamps, 2001), and trade‐offs related to mortality commonly involve plastic behavioral responses, as they reflect the need to circumvent plant defenses (Ferreira, 2017) as well as to hide or to eat less in order to avoid exposure to natural enemies (Agrawal et al., 2020; Bernays, 1997). An increase in larval development time leads to greater exposure to natural enemies and abiotic conditions, and thus higher risks for the larvae (Bernays, 1997; Schoonhoven et al., 1998; White et al., 2008), and the beginning of the reproductive period is delayed. Moreover, the size of adult *D. gilippus* larvae that used *C. procera* as a host was significantly larger than those that fed on *A. curassavica*. This difference is due to a higher proportion of male *D. gilippus* that were reared on this host compared with *A. curassavica* and not to host plant features, as males of this species appear to be slightly larger than females (PPS Ferreira, personal observation). Malcolm and Slager (2015) also found this dimorphism in field individuals of *D. erippus*. However, since there was no difference in the sex ratio of *D. erippus* reared on both hosts, no differences in body size between host plants were found in this regard.

Our field experiment indicated the existence of multiple factors that may control Danaini populations. These factors may act differently according to the host plant used in the larval stage, affecting life history traits of the herbivores in different ways. The hypothesis that bottom‐up pressures would predominate when *D. erippus* uses the exotic host plant has not been corroborated, since the "host plant" factor alone did not reduce larval survival significantly. Because *C. procera* is found in areas of the Restinga ecosystem, where *A. curassavica* is not expected to occur, the field experiment was conducted in two localities in the state of Rio de Janeiro. As a consequence, geographical location and host plant species might be confounding factors. Our laboratory experiment, in which both host plants were offered to larvae under the same controlled conditions, addressed this issue.

Larvae assigned to the bagged treatment showed a significant increase in survival on both hosts. Since early instars are small in size and limited in movement compared with later instars, physical aspects of the environment, including host plant traits and microclimatic conditions, may be critical to survival (Zalucki et al., 2002). As for microclimatic conditions, no significant differences between treatments were found for temperature and humidity of bagged and nonbagged leaves (Appendix S1). Wind intensity and precipitation, which differ in the localities where the experiments were conducted, were not recorded. Some studies point out that rainfall is one of the major factors causing mortality in eggs and newly hatched larvae (e.g., Caldas, 1992; Harcourt, 1966; Rahman et al., 2019). In this context, larval protection against natural enemies also prevents the larvae falling off the host plant. Additionally, the model that best explained the data shows an additive effect between bottom‐up and abiotic pressures determining larval survival under field conditions. As pointed out by Zalucki et al. (2002), factors affecting larval mortality may interact through synergism or antagonism. Thus, abiotic factors can exacerbate bottom‐up pressures, adversely affecting larval survival. Field studies on survival of monarch larvae on milkweeds that differ in the volume of latex produced or using intact versus severed milkweed leaves have shown that, in general, latex negatively affects larval survival (Zalucki, Brower, et al., 2001; Zalucki & Malcolm, 1999; Zalucki, Malcolm, et al., 2001). However, these studies did not disentangle latex effects from abiotic factors on larval survival. Our data strongly suggest that the high incidence of sunlight and wind can exacerbate the effects of latex exudation of *C. procera* on *D. erippus* survival.

No predation events were observed during the field experiment. Some studies have shown that predation has a great impact on the survival of immature insects (Cornell & Hawkins, 1995; Montllor & Bernays, 1993). However, it is possible that this factor is overestimated, especially for early instars whose absence is largely attributed to predation (Zalucki et al., 2002). In these cases, predation needs to be investigated in additional experiments (e.g., Bernays, 1997; Montllor & Bernays, 1993). The main natural enemies of *Danaus* spp. are the larvae and adults of *Harmonia* ladybug beetles (Koch et al., 2003) and *Polistes* wasps (Baker & Potter, 2020; McGruddy et al., 2020; Zalucki & Kitching, 1982, D Rodrigues and JR Trigo, personal observations) among predators, and tachinid flies among parasitoids (Dias, 1978; Oberhauser et al., 2007). It is important to note that these Danaini species are known for being unpalatable and having warning coloration (Brower & Moffitt, 1974; Endler & Map pes, 2004; Ritland, 1991), which may reduce predation rates in nature (Berenbaum & Miliczky, 1984; Rothschild et al., 1984).

In the regions where *C. procera* is native, this species is used as one of the main host plants by *Danaus chrysippus* (Linnaeus, 1758), the African queen butterfly (Brandes, 2005; Golestaneh et al., 2009; see Smith, 2014). According to Golestaneh et al. (2009), the larval development time of *D. chrysippus* feeding on *C. procera* leaves under similar controlled conditions to those in our study was about 4–5 days shorter than that of larvae of *D. erippus* and *D. gilippus* reared on this host (present study). This observation suggested to us that *D. erippus* and *D. gilippus* are not as adapted as *D. chrysippus* to using *C. procera* as a host plant, even though the former Danaini do use *C. procera* in the field (see Appendix S1 for field survey data). According to Pearse and Altermatt (2013), interactions between native herbivores and introduced plants can be predicted through the taxonomic relationships between exotic and native plants. The system studied here fits into the prediction that non‐native plants that belong to the same family as a native host plant are more likely to be colonized by native herbivores. In this sense, ecological fitting probably plays a role in the interaction between larvae of *D. erippus* and *D. gilippus* and *C. procera*, since it arises from the set of attributes that each species has at any particular moment, according to its previous evolutionary history (Agosta, 2006; Araujo et al., 2015; Janzen, 1985). With respect to the Danaus‐Apocynaceae system, many studies around the globe corroborate that monarchs, queens, southern monarchs, African queens, and the soldier butterfly consistently use milkweed species as host plants, regardless of region and plant origin, and are able to sequester specialized plant compounds and circumvent latex defenses (e.g., Ackery & Vane‐Wright, 1984; Becalloni, 2008; Fernandes et al., 2020; Malcolm & Slager, 2015; Smith, 2014; Zalucki, Brower, et al., 2001; Zalucki & Kitching, 1982; Zalucki et al., 2012; Zalucki, Malcolm, et al., 2001; this study and references above).

Preference and performance studies of *D. plexippus* oviposition using both native and exotic host plants showed a high preference of females for the native host; larval performance matches female preferences, in which exotic hosts ranged from poor to lethal resources (DiTommaso & Losey, 2003; Mattila & Otis, 2003). It would be worth employing a similar approach using the study system explored here to infer possible impacts of the introduction of *C. procera* on both *A. curassavica* and Danaini populations. Yoon and Read (2016) suggested, through a metanalysis, that exotic host plant species may decrease lepidopteran abundance by providing an ecological trap—that is, target for oviposition even being a poor larval resource. As landscapes become highly altered by anthropogenic environmental changes, it may be that exotic plants will be the unique host plant available to many herbivores. Thus, the occurrence of *D. erippus* and *D. gilippus* in the Restinga do Xexé appears to be due to the presence of *C. procera* and its use as a host plant, since *A. curassavica* is absent in that ecosystem and no alternative hosts were found (PPS Ferreira and D Rodrigues, personal observations). As a consequence, this exotic host can benefit these Danaini species by promoting an expansion of their distribution. In contrast, the use of *C. procera* in the field may adversely affect the survival of first instars as well as larval development time of the surviving larvae, as the latter spend a considerable amount of time and energy circumventing plant defenses when feeding on this host. These results indicate a cost–benefit relationship in the interaction between native herbivores and an exotic host plant. Finally, our study shows that field conditions may affect the survival of a native herbivore using a non‐native plant, when compared to an experiment conducted under controlled conditions. Therefore, experimental approaches in the field are a relevant tool to better understand the outcome of interactions between native insects and exotic plants.

## CONFLICT OF INTEREST

None declared.

## AUTHOR CONTRIBUTION

**Pedro Paulo da Silva Ferreira:** Conceptualization (supporting); Data curation (lead); Formal analysis (lead); Funding acquisition (supporting); Investigation (equal); Methodology (supporting); Project administration (supporting); Resources (supporting); Software (equal); Supervision (supporting); Validation (equal); Visualization (equal); Writing‐original draft (lead); Writing‐review & editing (supporting). **Daniela Rodrigues:** Conceptualization (lead); Data curation (supporting); Formal analysis (supporting); Funding acquisition (lead); Investigation (equal); Methodology (lead); Project administration (lead); Resources (lead); Software (equal); Supervision (lead); Validation (equal); Visualization (equal); Writing‐original draft (supporting); Writing‐review & editing (lead).

## DATA AVAILABILITY STATEMENT

All data are available via the Dryad data repository (https://doi.org/10.5061/dryad.3bk3j9kk2).

## Supporting information

App S1
