# Supplementary material for: Performance of Danaini larvae is affected by both exotic host plants and abiotic conditions
Source: Ecol Evol. 2021 Jun 23;11(14):9876–86. doi: 10.1002/ece3.7821 (PMC8293738; doi:10.1002/ece3.7821)
Supplement: Supplementary file 1 — App S1 [file ECE3-11-9876-s001.docx]

**Appendix S1**

**Electronic supplementary material**

Use of *Calotropis procera* and *Asclepias curassavica* by *Danaus* spp. DE: *Danaus erippus*; DG: *Danaus gilippus*. Data from Restinga do Xexé (Campos dos Goytacazes/RJ) and REGUA Reserve (Cachoeiras de Macacu/RJ), respectively.

| Host plant | Individuals | Sample | | | | Mean ± SE |
| --- | --- | --- | --- | --- | --- | --- |
|  |  | 1^st^ | 2^nd^ | 3^rd^ | 4^th^ |  |
| *Asclepias curassavica* | Plants | 30 | 30 | 30 | 30 | 30.00 ± 0.00 |
|  | Eggs | 20 | 2 | 3 | 31 | 14.00 ± 7.01 |
|  | Larvae (total) | 15 DE  3 DG | 19 DE | 1 DE  1 DG | 12 DE | 12.75 ± 3.90 |
|  | Plants | 16 | 23 | 21 | 20 | 20.00 ± 1.47 |
|  | Eggs | 5 | 0 | 0 | 5 | 2.50 ± 1.44 |
| *Calotropis procera* | Larvae (total) | 17 DE | 1 DE  1 DG | 0 | 2 DE | 5.25 ± 3.95 |
|  | Adults (sightings) | 3 DE | 0 | 0 | 2 DE | 1.25 ± 0.75 |
|  | Herbivory (%) | 6 – 12 | 1 – 6 | 1 – 6 | 6 – 12 |  |

**Methods**

Field surveys were performed in order to examine the use of the exotic host *C. procera* by *D. erippus* and *D. gilippus*. The survey consisted of an area of ​​5000 m² (50 m wide/100 m long) predominantly colonized by *C. procera.* Five 100 meter-long transects were established; transects were placed 10 m apart from each other. All *C. procera* plants touched by transects were sampled (N = 16). Eggs and larvae found on the selected plants were counted, as well as adults seen flying around the study area. Larvae were classified according to Danaini species and instar (species identification is not possible at the egg stage). In addition, the Herbivory Index was estimated according to the method established by Dirzo & Domínguez (1995). The study area is located in the Restinga do Xexé, Campos dos Goytacazes, Rio de Janeiro State (21º 59' 58.03" S, 40º 59' 06.85" W; 3 m a.s.l.). Surveys were conducted in June 2015, September 2015, December 2015 and March 2016.

In order to compare the use of *C. procera* by *D. erippus* and *D. gilippus* with the native host plant *A. curassavica*, we compiled data from eggs and larvae of both Danaini species collected from a population of this host located in the REGUA Reserve, Cachoeiras de Macacu municipality, Rio de Janeiro State (22º 27’ 10.30’’ S, 42º 46’ 13.05 W; 34 m a.s.l.). Sampling occurred in January, April, July and October 2014.

**References**

Dirzo R, Domínguez CA (1995) Plant-herbivore interactions in Mesoamerican tropical dry forests. In: Bullock SH, Medina E, Mooney HA (eds) Seasonally dry tropical Forests. Cambridge University Press, Cambridge, pp 304─325


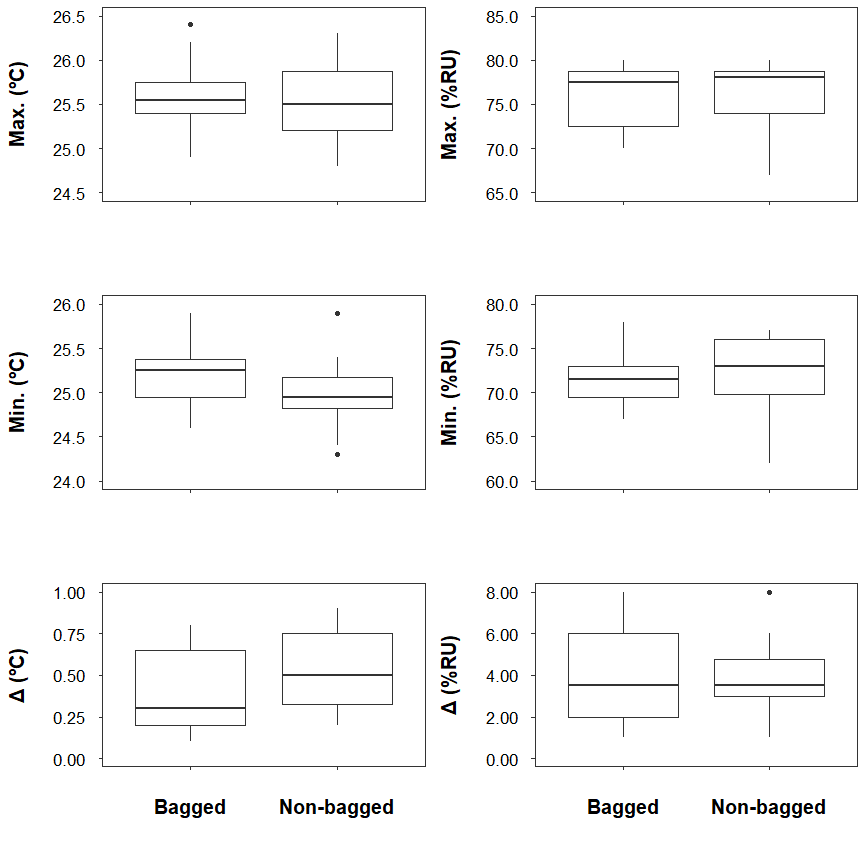


Comparison of air temperature (left panels) and relative humidity (right panels) between bagged and non-bagged leaves of *C. procera* (median, lower and upper quartiles and maximum scores). Dots above and below bars denote outliers. Δ denotes the differences between maximum (Max.) and minimum (Min.) values. No significant differences were found between treatments (Paired Student’s t-test, *P* > 0.05)
